# miEAA 2023: updates, new functional microRNA sets and improved enrichment visualizations

**DOI:** 10.1093/nar/gkad392

**Published:** 2023-05-13

**Authors:** Ernesto Aparicio-Puerta, Pascal Hirsch, Georges P Schmartz, Fabian Kern, Tobias Fehlmann, Andreas Keller

**Affiliations:** Clinical Bioinformatics, Saarland University, 66123 Saarbrücken, Germany; Clinical Bioinformatics, Saarland University, 66123 Saarbrücken, Germany; Clinical Bioinformatics, Saarland University, 66123 Saarbrücken, Germany; Clinical Bioinformatics, Saarland University, 66123 Saarbrücken, Germany; Helmholtz Institute for Pharmaceutical Research Saarland (HIPS), Helmholtz Centre for Infection Research (HZI), Saarland University Campus, 66123 Saarbrücken, Germany; Clinical Bioinformatics, Saarland University, 66123 Saarbrücken, Germany; Clinical Bioinformatics, Saarland University, 66123 Saarbrücken, Germany; Helmholtz Institute for Pharmaceutical Research Saarland (HIPS), Helmholtz Centre for Infection Research (HZI), Saarland University Campus, 66123 Saarbrücken, Germany

## Abstract

MicroRNAs (miRNAs) are small non-coding RNAs that play a critical role in regulating diverse biological processes. Extracting functional insights from a list of miRNAs is challenging, as each miRNA can potentially interact with hundreds of genes. To address this challenge, we developed miEAA, a flexible and comprehensive miRNA enrichment analysis tool based on direct and indirect miRNA annotation. The latest release of miEAA includes a data warehouse of 19 miRNA repositories, covering 10 different organisms and 139 399 functional categories. We have added information on the cellular context of miRNAs, isomiRs, and high-confidence miRNAs to improve the accuracy of the results. We have also improved the representation of aggregated results, including interactive Upset plots to aid users in understanding the interaction among enriched terms or categories. Finally, we demonstrate the functionality of miEAA in the context of ageing and highlight the importance of carefully considering the miRNA input list. MiEAA is free to use and publicly available at https://www.ccb.uni-saarland.de/mieaa/.

## Graphical Abstract

**Graphical Abstract:**
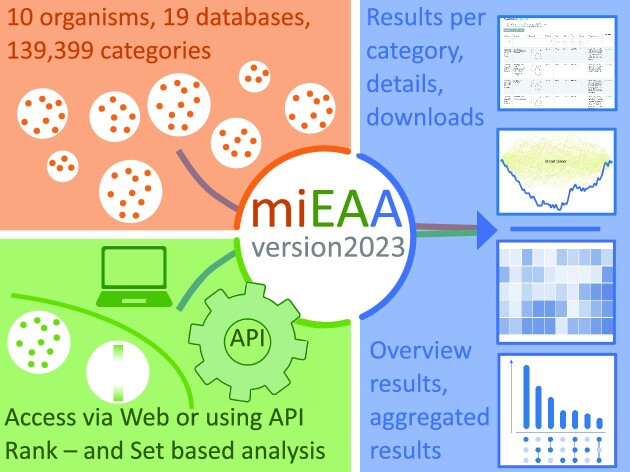

## INTRODUCTION

MicroRNAs (miRNAs) are a class of small non-coding RNAs of about 21–23 nucleotides that play a crucial role in post-transcriptional regulation of gene expression, mainly by binding to 3’UTR regions in target mRNAs to reduce or prevent translation (1). As one of the most studied non-coding RNAs, miRNAs have been described to be involved in virtually all biological processes (1), including pathologies such as cancer (2) or Parkinson's disease (3), and their expression correlates with aging (4). Their deletion in experimental models frequently leads to severe developmental consequences, which is not surprising considering that as much as 60% of all coding genes are potentially regulated by one or several miRNAs (5). Although they typically target more than one gene in the same pathway to regulate its activation (6), each miRNA gene can interact with several hundred transcripts spreading across distinct cell functions (7). This intricate network complicates straight-forward evaluation of the processes potentially affected by alterations or expression changes in a particular set of miRNAs. In this context, enrichment analysis has become a key tool to provide useful global insights into miRNA research, particularly in hypothesis-free setups which are a characteristic of NGS-driven studies.

Enrichment analysis is applied to sets of genes or miRNAs, frequently downstream of differential expression, to interpret and understand the functional aspects of an experiment. This process is necessary to extract relevant information from large lists of genes or miRNAs that would be infeasible to annotate manually. Some tools are specifically tailored to work with lists of miRNAs, either by direct annotation of miRNA genes and their mature transcripts or indirectly by providing enrichment analysis on the corresponding miRNA targets. One prominent example of the former approach is TAM (8), a webserver that relies on direct annotation of miRNAs from the Human MicroRNA Disease Database (HMDD) (9) and their own literature curation to include functional and disease-associated terms. In a more recent work, Cui *et al.* also introduced importance-based weighing of miRNAs to annotate in an essentiality-informed manner (10). Other highly used tools like DIANA-miRPath (11) and miRWalk (12) indirectly annotate miRNAs by deriving Gene Ontology and pathway terms from their target genes, although this approximation is not without caveats (13). Additionally, many mainstream gene-centric enrichment webservers like GeneCodis (14) or DAVID (15) have also included a miRNA module to accept miRBase identifiers as input. Finally, a third group of tools combine both strategies to include direct and indirect annotations in an effort to achieve consistent miRNA enrichment analysis on an augmented repertoire of terms obtained from gene annotations. In that respect, miEAA (16) stands out by allowing mature and precursor identifiers from 10 different species as input, compiling a total of 139 399 terms from 19 data sources. Furthermore, its API and command-line implementation make it very flexible and attractive to use in pipelines and other automated high-throughput applications where a graphic interface is not needed.

Rapidly developing knowledge on miRNAs, new databases that store respective information and updates in repositories that are included in the miEAA data warehouse call for regular updates and improvements. In this article we present the most recent version of miEAA, our miRNA Enrichment Analysis and Annotation framework based on GeneTrail (17). The current update is focused around further expanding the miRNA sets available in the tool. Most notably, the new additions include high confidence miRNAs and their evolutionary origin obtained from MiRGeneDB (18), tissue-specific and alternatively processed miRNAs from isomiRdb (19), and cell type-specific miRNAs determined from the human cellular microRNAome (20). Moreover, previously available datasets have been updated to reflect their latest additions. Finally, new plots have been designed to help users achieve better understanding of the processes underlying the provided miRNA list as well as of the interaction among the enriched terms or categories. MiEAA is free to use and publicly available at: https://www.ccb.uni-saarland.de/mieaa/.

## MATERIALS AND METHODS

### Web server implementation

The miEAA webserver was implemented in Django (v2.2), a web framework based on python (v3.7) and deployed using Docker containers. MiRNA sets are stored as Gene Matrix Transposed (*.gmt) files sorted by species and category. The frontend implementation relies on Bootstrap (v4.4.1) for styles and Font Awesome (v5.12.0) for icons, dataTables (v1.10.19) and jQuery (v3.3.1) for functionality and table display, plus Highcharts (v7.2.1) and plotly (v5.13) for interactive visualizations. As already implemented in the previous release, miEAA supports API querying using the Django REST framework. Lastly, jobs are internally scheduled using celery.

### New datasets integrated

The current update introduces new data from three resources: MiRGeneDB, isomiRdb and the human cellular microRNAome. From MiRGeneDB (v2.1) we have retrieved *bona fide*, high confidence, mature and precursor microRNA identifiers as well as the corresponding sets comprising locus or family of origin for the supported species (*bta, cel, dme, dre, gga, hsa, mmu, rno*). We used isomiRdb to calculate tissue-specific miRNAs in human, considering as such miRNAs with TSI values > 0.75. We also compiled sets of miRNAs that are confidently modified by TUTases or display alternative processing by DROSHA. Finally, we calculated cell-specific mature microRNAs from the human cellular microRNAome in the same fashion described for tissue-specific miRNAs. We lifted-over expression values to *bta*, *mmu* and *rno* using MiRGeneDB identifiers for orthology conversion. For example, hsa-miR-22–3p was converted to bta-miR-22-3p since they share the same MiRGeneDB identifier. External datasets previously present in miEAA including mammal ncRNA–disease repository (MNDR v3)(21), miRTarBase (v9.0) (22), RNAlocate (v2.0) (23), Gene Ontology (24) and KEGG (25) were updated following new releases using our Snakemake pipeline as described previously (26).

### Results visualization

In addition to the already available visualizations, namely term word-cloud and heatmap of enrichment *P*-values, several new graphs have been implemented to provide users with a better overview of the most significant terms, the *P*-value distribution across categories and the overlap between different sets. Several dropdown menus have also been implemented to allow subselection of categories of interest.

***Bar chart*.** This graph allows the user to list enriched terms sorted by significance, number of hits or Observed/Expected ratio and represent the values as bars.

***Categories summary and categories p-values*.** These two plots show the count of enriched terms per category and boxplots of their p-values, respectively. Besides a general overview of all the enriched terms, they enable quality control to discard that all enriched terms belong to the same category or whether p-values are uniformly distributed across categories.

***Upset plot*.** This chart can be used to visualize the intersections between different enriched sets, which can reveal a core set of miRNAs that are present in multiple relevant categories. This could either hint a possible interesting network, target of further downstream investigation, or reveal the existence of an overlapping subset of miRNAs that leads to the co-detection of these terms as significantly enriched when this subset is present in the input list.

### Use case dataset

From the original non-coding RNA atlas in aging publication (27), we downloaded the supplementary table containing correlation coefficients. For each tissue, Spearman rank correlation with age was calculated using every miRNA expressed over 1 rpmm in at least 10% of the samples in that tissue. Overrepresentation analysis (ORA) was performed using positively correlated miRNAs (*r* > 0.5). MiRNA Set Enrichment Analysis was performed on miRNAs sorted in decreasing order by Spearman rank correlation coefficient (Supplementary Table 1). For aggregated analysis, miRNAs considered for overrepresentation in each tissue were intersected and used to perform ORA on miRNAs positively correlated in at least 3, 4 and 5 different tissues.

## RESULTS

### Updates and new functionality facilitate a broader application spectrum

The latest release, miEAA 2023, focuses on two main objectives: improving the underlying data warehouse and providing new graphical representations to facilitate a rapid interpretation of results across different categories. Additionally, we have improved the backend functionality, making it possible to analyse hundreds of requests in a more convenient manner using a more functional API.

**Figure 1.**
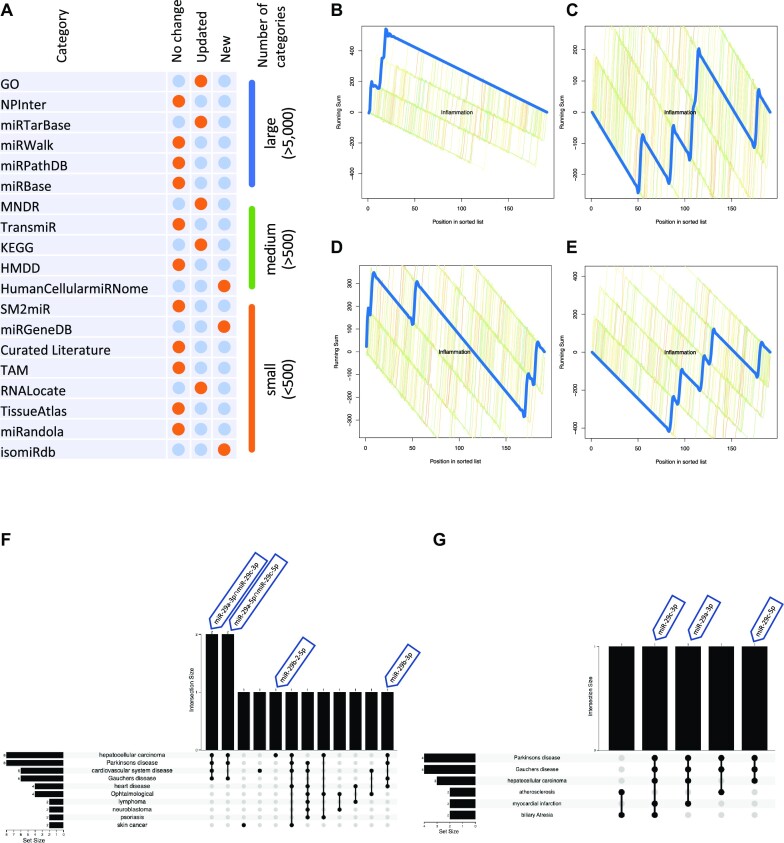
(**A**) Databases and repositories included in miEAA. The first column represents the name of the database. The following three columns highlight whether the database is unchanged, updated, or newly added by orange bubbles. The last column groups the repositories in the size. Here, size only referrers to the number of categories added to miEAA. (**B**) Running sum for MNDR term inflammation in brain tissue (**C**) Running sum for MNDR term inflammation in brown adipose tissue (**D**) Running sum for MNDR term inflammation in marrow adipose tissue (**E**) Running sum for MNDR term inflammation in subcutaneous adipose tissue (**F**) Upset plot of the 10 most significantly enriched MNDR diseases for age-associated miRNAs in at least three tissues. (**G**) Upset plot of the 6 significantly enriched MNDR diseases for age-associated miRNAs in at least four tissues.

The data warehouse is a core strength of miEAA, integrating numerous resources developed by us and others. In the previous version, we included 16 different databases. In the current release, we have added three more repositories and updated the existing ones (Figure 1A). To facilitate the understanding, we grouped the 19 repositories we used in miEAA into three sets based on the number of categories they add: very large repositories with at least 5000 categories, medium-size repositories with at least 500 categories, and small repositories with fewer than 500 categories. Of note, our size classification is not based on the database's overall size or functionality but on the number of categories added to miEAA from the respective resources. Interpreting the databases within each category provides evidence that miEAA 2023 describes updates of the large and medium-sized databases while the newly added resources are medium-sized to small (Figure 1A), in line with the current trend towards more specific and functional miRNA research.

We have observed that users' queries are becoming increasingly complex in terms of their number and frequency, which is further aggravated by the growing number of terms available, particularly in human. To address this challenge, we have added functionality for aggregated analysis, allowing users to interpret the results of all categories in a single run. Upset plots were found to be among the most useful representations we explored during the testing phase because they allow interactive comparisons of an arbitrary number of intersections. This way, a user can identify relevant sets of miRNAs that are shared across several genes, pathways, or diseases. The updated data sets, the new repositories supported and the enhanced functionality to interpret significant categories and their overlap in context greatly improve the application scope of miEAA.

### Ageing research as use case to demonstrate the performance of miEAA

To demonstrate the functionality of miEAA, we analysed the enrichment from miRNA expression patterns in ageing mice as use case. In short, this study reported several miRNAs that correlated with ageing in different tissues after profiling 771 samples employing miRNA-seq (27). We first performed Overrepresentation Analysis using the list of positively correlated miRNAs for each tissue. No general trend could be observed after individual assessment of each list, besides the occasional appearance of Parkinson's disease, which is by definition associated with age. Nevertheless, some interesting terms were significantly enriched for specific tissues. For instance, the Gene Ontology term ‘Negative regulation of fat cell differentiation’ was significantly overrepresented for marrow adipose tissue and *Hdac4*, a gene involved in bone and muscle development, was overrepresented in miRNAs from bone. In fact, some studies have proposed the usage of different *Hdac4* inhibitors as potential anti-ageing therapy. We then performed miRNA Set Enrichment Analysis on the miRNAs ranked for each tissue by their correlation coefficient and identified some interesting enrichments. For instance, we found the disease term inflammation obtained from the mammal ncRNA–disease repository (MNDR), to be significantly enriched in the brain (*P*-value = 6.04e–4, Figure 1B), an organ chronically affected by systemic inflammation (28). Although inflammation is a healthy physiological response, it tends to become chronic with age in a process where the influence of the adipose tissue is well known (29). Surprisingly though, we did not find this term to be significantly enriched in subcutaneous, brown or marrow adipose tissue (Figure 1C–E). Additionally, other disorders that typically increase with age such as diabetes or metabolic disease were also not present in these results.

We next proceeded to analyse the enrichment of the overlapping miRNAs in an aggregated manner (i.e. correlated with aging in more than *n* tissues) to obtain global insights. For the 30 miRNAs correlated in at least 3 different tissues, 171 entries were detected as significantly overrepresented. The analysis revealed several significant disease terms that are more frequent in aged individuals like sarcopenia, sensorineural hearing loss, heart disease, cardiovascular disease, Parkinson's disease, and several types of cancer, which is not surprising considering the input miRNAs positively correlated with age in several tissues. Using the interactive UpSet plot (Figure 1F), we identified that at least one or more miR-29 family members (miR-29a-3p, miR-29c-3p, miR-29a-3p, miR-29c-5p, miR-29a-5p, miR-29b-2-5p) were present in every one of the five most significantly enriched diseases. The original study already described and validated a prominent role of this miRNA family in the ageing process, particularly miR-29a-5p, which was positively correlated with age in eight different tissues. This work also found a potential EXOmotif present in the miR-29 family (30), which is coherent with the detected overrepresentation of miRNAs located in Extracellular Vesicles according to RNAlocate (*P*-value = 0.0015). After restricting our input list to age-associated miRNAs in at least four tissues, the enriched entries were reduced to 43. Among these, we could no longer find most diseases from the previous analysis except for Parkinson's disease and hepatocellular carcinoma. Notably, five out of the six significantly overrepresented diseases have one or several hits from the miR-29 family (Figure 1G). After requiring 5 different tissues, this effect further increased and only Parkinson's disease remained from the previously described set. In view of the importance of the miR-29 signal in the input list and to explore bias in the results due to their inflated presence, we decided to collapse all miRNAs belonging to this family into miR-29a-3p and miR-29c-5p, the most frequently correlated from each precursor arm. Since annotations for these miRNAs will highly overlap, particularly those derived from computational predictions based on the seed sequence, we expected this would reduce its influence to some extent. By removing just these few miRNAs from the list, the total amount of overrepresented terms detected dropped from 171 to 22. Despite the strict filtering, many terms previously identified like Hyperalgesia or Parkinson's disease remained overrepresented so the enrichment in at least some categories goes beyond the influence of the miR-29 family.

In summary, our use case exemplifies the functionality of the current version of miEAA in providing a sensible overview of the processes potentially connected to given sets of miRNAs. These results also highlight how small changes in the input list can have important consequences in the enrichment analysis, calling for careful consideration of the inclusion criteria or parameters.

### The enlarged repertoire of sets provides new insights

Besides the incorporated visualizations, designed to support results interpretation, there is also value in the newly generated and updated datasets. To illustrate this, we reanalysed previous studies that relied on miEAA and explored insights and hypothesis that can only be derived using the most recent version. For instance, a study that proposed miRNA-based biomarkers to diagnose hypertrophic cardiomyopathy identified several enriched KEGG entries associated to the list of altered miRNAs such as several lipid and amino acid metabolic pathways (31). Using the new version, we identified 49 new significantly enriched terms, including miRNAs that display TUT4/7-uridylated isoforms. Interestingly, defective miRNA uridylation has previously been linked to myotonic dystrophy which can lead to hypertrophic cardiomyopathy (32). We also compared the previous and current output from a different study that described how the miRNA cargo of extracellular vesicles (EVs) derived from mesenchymal stromal cells reduced inflammation in a dry eye disease model (33). The authors examined the 10 most abundant miRNAs in EVs using miEAA to dissect the signalling processes that might be driving the observed effects and concluded that these miRNAs targeted several important immune-related pathways like NF-κB. Along the same lines, we found one new enriched target gene added from the latest miRTarBase release, *AKT1*, which is highly expressed in immune cells and participates in different inflammatory pathways (34,35). Finally, we compared the enrichment results obtained from dysregulated lung miRNAs in a mouse *Cryptococcus neoformans* infection model (36). The current version revealed several enriched GO terms associated to defence responses (GO0002357:defense response to tumor cell, GO0051607:defense response to virus, GO0042742:defense response to bacterium, GO0050830:defense response to Gram-positive bacterium) whereas the previous release only found one (GO0051607:defense response to virus). Even though *C. neoformans* is not included among neither of the above-mentioned pathogenic agents, it can be assumed that it would elicit a cellular reaction similar to the others. In the original work, the authors analysed the enriched KEGG pathways to eventually conclude the activation of similar responses. Now, the same conclusion can be directly derived from the input miRNA list.

## DISCUSSION

The version of miEAA presented here offers a complete and comprehensive miRNA enrichment tool considering the broad compilation of direct and indirect terms obtained from a variety of updated or newly added resources; the incorporated visualizations such as Upset plot, which enables the exploration of set intersections; and the API service for automated high-throughput querying using ORA and GSEA. We have showcased some of the new functionalities with an example and by comparing the enrichment results of three studies to the previous version, warning about the consequences of overrepresenting miRNAs from the same family in the input and highlighting the implications of even very slight changes in the list. In light of this observation, the convenience of the new visualizations to spot overlapping sets of miRNAs across different enriched categories becomes apparent.

Admittedly, the current term repertoire expansion has only been possible by orthology conversion of human entries and derivation from target gene annotations. These transfer approaches are limited by the existence of orthologous miRNAs, besides other shortcomings previously described, and may eventually lead to false positive entries. Although ideally most terms should be based on species-specific direct annotations extracted from literature review, this can be challenging if not infeasible for many species, particularly because it is harder to automate, and far more research is published on human compared to other species. For instance, *Gallus gallus* has 30 times fewer entries than human and most categories come from miRBase, miRTarBase and KEGG, since other resources do not support this species. In this context, robust methods for indirect annotation become a great supplement to perform enrichment analysis or even become a complete replacement for situations where a better alternative is not available.

Rapid changes in miRNA research and growing numbers of user requests call for regular web server releases. One example of new functionality that will be added next is cross-dataset comparison of results (e.g. useful for time series data), cross-species comparison of the results (e.g. comparing results for a cancer in humans and mouse models) and inter-species regulation (e.g. small RNAs from bacteria targeting eukaryotic genes (37)). These examples demonstrate that despite 8 years of development, a growing body of new analysis functionality is required to keep track with developments in the miRNA research field. Current and upcoming features position miEAA as a tool that supports the functional and mechanistic understanding of miRNA roles. MiEAA however already stands out in this aspect as a versatile solution allowing users to restrict the categories used for enrichment analysis to direct or indirect depending on the target species or their specific needs and relying on useful visualizations that can help spot biologically interesting subsets and discerning them from potentially misleading annotation artifacts.

## DATA AVAILABILITY

miEAA 2.1 is freely available at https://www.ccb.uni-saarland.de/mieaa/. No login is required. All datasets included in the tool are available in the downloads page.

## Supplementary Material

gkad392_Supplemental_File
